# Linking Empowering Leadership and Employee Work Engagement: The Effects of Person-Job Fit, Person-Group Fit, and Proactive Personality

**DOI:** 10.3389/fpsyg.2018.01304

**Published:** 2018-07-31

**Authors:** Di Cai, Yahua Cai, Yan Sun, Jinpeng Ma

**Affiliations:** ^1^School of Management, Shandong University, Jinan, China; ^2^Department of Human Resource Management, Shanghai University of Finance and Economics, Shanghai, China

**Keywords:** empowering leadership, work engagement, person-job fit, person-group fit, proactive personality

## Abstract

Based on person-environment fit theory, this study examined the effects of empowering leadership on employee work engagement. We also investigated the mediating mechanism of person-job fit and person-group fit. In addition, we explored employee proactive personality’s moderating role between empowering leadership and the above two kinds of fit, and then the set of indirect effects. Using a survey sample of 6179 employees from a technology company in China, we found that empowering leadership has a positively indirect influence on employees work engagement though person-job fit and person-group fit. Further, moderated mediation analysis revealed proactive personality augmented empowering leadership direct effect on person-job fit and person-group fit and indirect effect on work engagement. Theoretical and practical implications were also discussed.

## Introduction

Work engagement is conceptualized as a positive, fulfilling and work-related state of mind (Schaufeli and Bakker, 2004, 2010). Recently, the study of employee work engagement has gained significant attention, particularly with researches showing its relevance for organizational outcomes (Jorge Correia de Sousa and van Dierendonck, 2014). For example, researches have revealed that work engagement is positively associated with organizational commitment (Hakanen et al., 2006), job satisfaction (Lu et al., 2016), and job performance (Bakker and Bal, 2010). Thus, it is theoretical relevant to explore its antecedents (Bakker, 2011). Among its several possible antecedents, leadership has been identified as an important driver of work engagement (Carasco-Saul et al., 2015), specific leadership behaviors which are found to enhance work engagement include transformational leadership (Zhu et al., 2009; Aryee et al., 2012), servant leadership (Jorge Correia de Sousa and van Dierendonck, 2014), authentic leadership (Walumbwa et al., 2008; Alok and Israel, 2012), charismatic leadership (Babcock-Roberson and Strickland, 2010) and empowering leadership (Tuckey et al., 2012). However, few studies have explored the relationships between empowering leadership and work engagement in the Chinese context. Therefore, the major goal of our research was to build and test theory that links empowering leadership with work engagement, and explore the mechanisms and boundary condition.

Besides neglecting the relationship between empowering leadership and work engagement, few previous researches has investigated the mediating mechanism. One notable exception is Tuckey et al. (2012), they used Job Demands-Resources framework to investigate cognitive demands and cognitive resources as mediators between empowering leadership and work engagement. This study draws on the person-environment fit framework, which highlights that the compatibility between person and environment made positive effect on desirable work-related outcomes such as voice behavior and engagement (Kristof-Brown et al., 2005; Chen et al., 2014). Among the many environment cues which can induce follower’s fit perception, leadership are among the most proximal and influential factors (Salancik and Pfeffer, 1978; Kristof-Brown et al., 2005), thus it is theoretical bold to invoke person-environment fit framework to link empowering leadership and work engagement. Besides, previous work suggested that various person-environment fit perceptions play a pivotal role in the process of leadership and engagement (Zhang and Bartol, 2010; Hsieh and Wang, 2015). This study invokes person-job fit, a task-related fit perception, and person-group fit, a relational-related fit perception to delineate how empowering leadership affects work engagement. Research has revealed that among all types of fit, person-job fit and person-group fit make the most significant effect on job performance (Kristof-Brown et al., 2005). Several studies have provided predictive validity evidences of person-job fit (Kristof-Brown et al., 2014). For example, Enwereuzor et al. (2016) investigated the mediating role of person-job fit in explaining the relationship between transformational leadership and work engagement. Thus, we argue that employees’ perceptions of job and group fit will jointly fulfill the psychological needs that the job is self-determined and they are supported by other team members, and then enhanced work engagement (Vera et al., 2016). By doing this, this study responds to recent calls from fit researchers by focusing on the jointly impact of both task and relationship perceptions of fit (Kristof-Brown et al., 2005).

In addition, little research has considered the boundary conditions between empowering leadership and work engagement. Leadership did not exist in a social vacuum, and instead is a social process evolved around the interactions between leaders and followers (Zhu et al., 2009). Also, trait activation theory (Christiansen and Tett, 2013) stated that fit perceptions derived mainly from the interactions between contextual factors and individual differences. Thus, follower’s individual differences such as proactive personality have a vital effect on how they response to leader’s behaviors (Zaccaro, 2012; Newman et al., 2017). Meanwhile, Kristof-Brown et al. (2005) also indicated that there was urgent need to explore the moderation effect of personal and situational characteristics among the fit research field. Practically, it is important for managers to understand in which situation empowering leadership can enhance followers’ work engagement (Tuckey et al., 2012). To address the gap, we chose proactive personality as the moderator between empowering leadership and work engagement. Hence, the third purpose of this study is to examine proactive personality’s moderating effect on the relationship between empowering leadership and work engagement.

Anchoring in person-environment fit framework, this study makes three contributions to the current literatures. First, it further extends the literature of work engagement by theorizing empowering leadership as its antecedent, we move beyond Tuckey et al. (2012)’s work, which link empowership leadership and work engagement by using Job Demand-Resource model. Second, the study also extends our understanding of the underlying mechanisms of the relationship between empowering leadership and work engagement under person-environment fit theory. Furthermore, in order to provide more nuanced understanding of the process by which empowering leaders engender work engagement, this study also investigate the boundary conditions from person-environment interaction perspective to identify which followers may benefit more from empowering leaders. This collection of insights will allow organizations to better improve employee work engagement and deploy empowering leaders to the maximum benefit of the organization.

## Theory and Hypothesis

### Empowering Leadership and Employee Work Engagement

Work engagement is defined as a positive and work-related state of fulfillment which is characterized by behaviors of vigor, dedication and absorption (Schaufeli and Bakker, 2004, 2010). Researches have revealed that high levels of work engagement had positive effects on various outcomes, such as better psychological health (Schaufeli et al., 2006; Bakker and Bal, 2010) and fewer psychosomatic complaints (Schaufeli and Bakker, 2004). Several evidences also link engagement to superior work performance (Bakker and Bal, 2010) and other beneficial outcomes for employers, including organizational commitment (Hakanen et al., 2006), organizational satisfaction (Hakanen and Schaufeli, 2012; Macey and Schneider, 2008) and proactive behavior (Salanova and Schaufeli, 2008).

Given its importance, it is of theoretical importance to explore work engagement’s antecedents. Among its many predictors, leadership is undoubtedly importance since organizations increasingly structured work into team-based (Kozlowski and Bell, 2003). Recent research indicates that different forms of leadership (e.g., transformational leadership, servant leadership, authentic leadership, charismatic leadership) are related to work engagement (Walumbwa et al., 2008; Babcock-Roberson and Strickland, 2010; Alok and Israel, 2012; Aryee et al., 2012; Jorge Correia de Sousa and van Dierendonck, 2014). However, there has been little research focusing on the effect of empowering leadership on work engagement. Empowering leadership has demonstrated its importance since it can provide increased sense of autonomy to employees (Zhang and Bartol, 2010; Tuckey et al., 2012). In addition, empowering leadership, as a form of relational leadership, involves sharing power that can enhance employees’ motivation and involvement in their work (Thomas and Velthouse, 1990; Kirkman and Rosen, 1999). Thus, there are sufficient reasons to expect the positive relationship between empowering and work engagement. We turn now to the main contribution of this study—an explanation of how empowering leadership influences work engagement.

Conger and Kanungo (1988) defined empowerment as a process that managers sharing power with followers. Wang et al. (2008) defined empowering leadership as behaviors that motivate followers to achieve excellent performance level. In order to further explore mechanism of empowering leadership on subordinates’ behaviors and attitudes in Chinese Context, Wang et al. (2008) further developed an indigenous measurement of empowering leadership. Empowering leadership behaviors have six dimensions, which are support for individual development, process control, delegation of authority, outcome control, participation in decision-making, and coaching for work (Wang et al., 2008).

Theoretically, we argue that empowering leadership is conducive to employee work engagement from two aspects. On the one hand, empowering leadership can fuel intrinsic motivation to stimulate work engagement. Empowering leader involves granting employees a fair amount of autonomy, which can make employees feel responsibility for their jobs and be motivated to achieve their goal (Vecchio et al., 2010). Specifically, delegation of authority and participation in decision-making can improve employees’ capacity for self-determination and employees’ feelings of mastery, which can enhance the employees’ motivation for work engagement (Stone et al., 2009; Zhang and Bartol, 2010; Deci et al., 2017). On the other hand, we propose that empowering leadership can provide employees with sufficient resources which enable follower to engage in their works (Bakker, 2011). State directly, by supporting for individual development and coaching for work, empowering leaders can help employees meet their basic need for competence and fulfill their work goals (Ryan and Deci, 2000; Tuckey et al., 2012). Further, empowering leaders can encourage employees to act proactively (Wang et al., 2008). Through these behaviors, employees can generate sufficient resources to handle job demands and feel more meaningfulness, which have positive effects on employee work engagement (Tuckey et al., 2012). In support of this, Kahn (1990) indicated that psychological meaningfulness was conducive to work engagement. Tuckey et al. (2012) also indicated that team level empowering leadership relates positively with follower work engagement, and the effect is mediated by cognitive resources. Therefore, we hypothesize the following:

H1: *Empowering leadership is positively related to employee work engagement.*

### The Mediating Role of Person-Job Fit and Person-Group Fit

#### Person-Environment Fit

Person-environment fit (P-E fit) can be defined as “the compatibility between an individual and a work environment” (Kristof-Brown et al., 2005, p. 281). Grounding in the Lewin’s field theory (Lewin, 1951), one of the key predictions of P-E fit theory is that individual performance are affected by the interaction between individual differences and organizational characteristics (Kristof-Brown et al., 2005; Seong et al., 2015). P-E fit research is generally focused on various characteristics of their work environment, such as their vocation (person-vocation fit), organization (person-organization fit), job (person-job fit), and group (person-group fit) (Kristof-Brown et al., 2005; Seong et al., 2015). Although there are conceptual level differences among various type of fit, and empirical study demonstrated their individually positive effect on employees’ behavior, there are few researches that examine their joint impact. In this study, we chose one task-related fit perception, named person-job fit, and one relational-related fit perception, named person-group fit. Among various types of relational fit, person-group fit has been demonstrated to be the key type which can predict employee performance, especially in the team context (Seong et al., 2015). Basically, this study examine two types of fit from two different perspectives, that is person-job fit based on task perspective and person-group fit based on relationship perspective, to explore their mediated effect between leadership and work engagement.

#### The Mediating Role of Person-Job Fit

Person-job fit (P-J fit) refers to the relationship between employee characteristics and job characteristics (Kristof-Brown et al., 2005). Edwards (1991) outlined a two-dimensional conceptualization of P-J fit consisting of needs-supplies (N-S) fit and demands-abilities (D-A) fit. N-S fit refers to the congruence between employee needs, desires, and preferences and the reward received for the job. D-A fit is the compatibility between job demands and employee’s knowledge, skills and abilities (Cable and DeRue, 2002). Prior studies have supported the effect of P-J fit on employee and organizational attitudes and behaviors (Warr and Inceoglu, 2012; Farzaneh et al., 2014; Kristof-Brown et al., 2014).

Based on person-environment fit theory, we argue that empowering leadership is positively related to person-job fit from the above two aspects. First, empowering leadership can improve employees’ need-supply fit (N-S fit). As a supportive leadership, an empowering leader focus on employees’ individual development, providing not only guidance on work but also the resources required to complete the job (Wang et al., 2008; Enwereuzor et al., 2016). Besides, the temperate control of the empowering leaders indicates that leader focus on the employees’ need at each stage from goal to outcome, thus promoting the sense of need-supply fit (De Beer et al., 2016; Gyu Park et al., 2017). Second, empowering leadership can improve employees’ demand-ability fit (D-A fit). The coaching and control for work from empowering leader may reduce employees’ role ambiguity (Wang et al., 2008), thereby enhancing their capabilities to fulfill job demand (Tuckey et al., 2012). Moreover, by delegating authority to employees, an empowering leader is likely to provide employees with trust and approval, which promotes employee belief that they have sufficient abilities and resources to get the job done (Ahearne et al., 2005), thus promoting the sense of D-A fit. Combining these logics, we propose that empowering leadership is positively related to person-job fit. Additionally, prior research already contended the positive relationship between person-job fit and employees’ work engagement (De Beer et al., 2016; Enwereuzor et al., 2016; Bui, 2017). According to the norm of reciprocity and social exchange theory (Cropanzano and Mitchell, 2005), employees are supposed to pay back when they got resources and support. State directly, employees will find their job worthwhile to when their needs have been satisfied and the demands of the job also match their knowledge, skills and abilities. Taken together, empowering leadership stimulates employee’s person-job fit, which in turn influences employee work engagement. Thus, we propose the following hypothesis:

H2: *Person-job fit mediates the relationship between empowering leadership and employee work engagement.*

#### The Mediating Role of Person-Group Fit

Person-group fit (P-G fit) is defined most broadly as “the compatibility between individuals and their workgroups” (Kristof, 1996, p. 7). Person-group fit could be conceptualized along supplementary fit and complementary fit (Muchinsky and Monahan, 1987). Specifically, supplementary fit occurs when a person possess a characteristic which supplements, embellishes their surrounding environment (Muchinsky and Monahan, 1987, p. 269). Meanwhile, complementary fit occurs when a “weakness or need of the environment if offset by the strength of the individual, and vice versa” (Muchinsky and Monahan, 1987, p. 271). P-G fit can happen in either case when one is similar to other organizational members on values (supplementary fit) or when one possesses job-relevant knowledge, skills, and abilities needed by their work group (complementary fit).

We invoke optimal distinctiveness theory (ODT) to delineate the process by which empowering leadership can fulfill follower’s fundamental psychological need, thus enhance person group fit. The ODT proposes that humans have two fundamental social needs. First, people desire to be socially included in a group, meaning that the similarity among them is emphasized (Leonardelli et al., 2010). Second, people also seek to identify themselves from other persons, meaning that individuals also value their unique characteristics (Leonardelli et al., 2010). We postulate that empowering leadership can enhance person-group fit. In terms of the need for belongingness, according to Arnold et al. (2000), the coaching behavior encouraging employees to solve problems, thereby providing them with opportunities to improve team cohesion and form collective team identification (Kasemsap, 2013). Second, empowering leadership creates opportunities for employees to participate in decision-making which promotes the chance for employees to engage in team work and create team-based commitment. Therefore, empowering leadership could satisfy follower’s belongingness need and thus enhance their supplementary fit.

With regards to the need for uniqueness, on one hand, when the leader gives followers opportunities to participate in decision making, employees have more opportunities to share their unique ideas and express personal suggestions (Wang et al., 2008). Under such circumstances, team members exert substantially influence on decision making, and employees might find their unique knowledge and skills are practically valuable to the team (Zhang and Bartol, 2010). On the other hand, the support and temperate control form empowering leader convey an important signal that leaders are open to employee’s involvement in key task related decisions, thus help employees to recognize their unique capabilities (Ahearne et al., 2005), by doing so, empowerment leadership can meet employee’s need for uniqueness, and thus promote complementary fit. Combining these logics, we propose that empowering leadership is positively related to person-group fit.

Based on self-determination theory, we argue that high person-group fit means employees’ social needs are satisfied and would lead to higher work engagement (Guan et al., 2011; Deci et al., 2017). Accumulating studies showed that under high person group fit, individuals are more willing to engage in their job (Kristof, 1996; Memon et al., 2014) Besides, employees who have unique knowledge, skill and ability which are coupled with their team’s demands may work more engaged to perform their jobs well (Tuckey et al., 2012). Given the above findings, we would expect person-group fit to mediate the relationship between empowering leadership and employee work engagement. Based on the above arguments, we develop the following hypotheses:

H3: *Person-group fit mediates the relationship between empowering leadership and employee work engagement.*

### The Moderating Role of Proactive Personality

Proactive personality is defined as an individual’s stable trait toward proactive behavior, which aimed at identify opportunities and act on them to affect the environment (Crant, 2000). Based on person-environment fit theory and its seminal work—field theory, we argue that people response differently to empowering leadership, and thus form different person-job fit and person-group fit based on the congruence between their personal characteristic and empowering leadership. Specifically, compared to more passive employees, people who are proactive show more initiative and they do not wait for opportunities, but actively seek information and take the initiative to solve problems and make meaningful change (Fuller et al., 2012; Ng and Feldman, 2013). Furthermore, they are more inclined to improve their circumstances by themselves rather than passively adapt to current circumstances (Crant, 2000; Bakker, 2011). In the workplace, proactive individuals are more likely to search for new ideas and information, obtain work support, pursue opportunities for self-improvement, and engage in work activities (Seibert et al., 1999).

We argue that proactive employees may be more likely to perceive high person-job fit and person-group-fit when their leader engage in empowering leadership behaviors than passive ones. Specifically, employees who have high proactive personality will typically perceive high person-job fit under empowering leader for several reasons. Prior study has shown that proactive people are more likely to identify opportunities and take initiatives (Crant, 2000). We argue that such employees can utilize opportunities provided by empowering leaders (Newman et al., 2017). Thus, employees high in proactive personality will perceive more N-S fit when their leaders giving support and delegate power (Wang et al., 2008). This should further lead employees to feel that their abilities match the job demands and become more engaged (Wang et al., 2017). Along the similar line, recent research suggests that employees with high proactive personalities are more willing to respond to positive leadership behaviors (Newman et al., 2017). For instance, Newman et al. (2017) demonstrated that proactive personality could strength the effects of servant leadership on organizational citizenship behavior.

We also proposed that proactive personality would strength the effect of empowering leadership on person-group fit. First, for high proactive personality employees, empowering leaderships coach the work and control the process can form consistent values with followers more easily which can enhance the complementary fit (Seong et al., 2015). Second, we expect that high proactive personality employees could perceive more supplementary fit under empowering leadership. Past research has established that employees high in proactive personality actively seek information, take the initiative to improve things and shape their own environment (Crant, 2000). Therefore, when empowering leadership creates opportunities for employees to participate in decision-making and delegate the authority, high proactive personality employees will perceive more trust and complementary fit. This leads us to the following hypotheses:

Hypothesis 4a Proactive personality will positively moderate the effects of empowering leadership on person-job fit: The relationship is stronger when proactive personality is high than low.

Hypothesis 4b Proactive personality will positively moderate the effects of empowering leadership on person-group fit: The relationship is stronger when proactive personality is high than low.

### An Integrative Moderated Mediation Model

Thus far, we have developed theoretical underpinnings for the mediating effect of person-job fit and person-group fit and the moderating effects of the proactive personality. That is, person-job fit mediates the relationship between empowering leadership and work engagement (Hypothesis 2). Person-group fit mediates the relationship between empowering leadership and work engagement (Hypothesis 3). Proactive personality moderates the positive relationship between empowering leadership and person-job fit (Hypotheses 4a), and the positive relationship between empowering leadership and person-group fit (Hypotheses 4b). The theoretical rationales behind the above hypotheses also suggest an integrative moderated mediation model. Specifically, employees’ proactive personality may moderate the indirect effect of empowering leadership on work engagement through person-job fit and person-group fit. The theorizing rationales behind Hypotheses 2, 3, 4a and 4b indicates that through augmenting or attenuating the association between empowering leadership and person-job fit, employees’ proactive personality affect the degree to which empowering leadership effects employee work engagement. Likewise, employees’ proactive personality, owing to their moderating influence on the link between empowering leadership and person-group fit (Hypotheses 4b), may also hold the potential of changing the indirect effect of empowering leadership on work engagement through person-group fit. Taking these predictions together, we propose two sets of integrative moderated mediation hypotheses:

Hypothesis 5a: Proactive personality moderate the indirect positive effect of empowering leadership on work engagement via person-job fit: The positive indirect effect is stronger when employees’ proactive personality is higher (vs. lower).

Hypothesis 5b: Proactive personality moderate the positive indirect effect of empowering leadership on work engagement via person-group fit: The indirect positive effect is stronger when employees’ proactive personality is higher (vs. lower).

**FIGURE 1:**
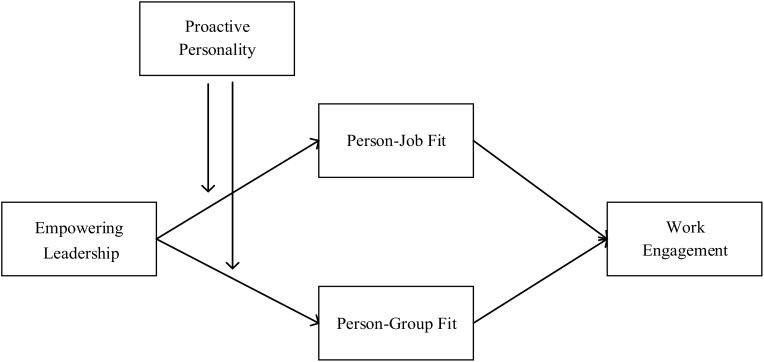
Concept model.

**Figure 1** depicts the conceptual model in this study.

## Materials and Methods

### Samples and Procedure

The participants in this study came from a single company, which was among the top 100 in the IT industry in China. An ethics approval was not required as per the author’s Institution’s guidelines and national regulations. The CEO of the company supported this research. For the participants, we sent online questionnaire which directly links to individual members. They were completely free to join or drop out the survey. Only those who were willing to participate were recruited. The cover page of the questionnaire lay out the study objectives, the voluntary nature of the survey, and an assurance of confidentiality to the participants. The consent of the participants was obtained by virtue of survey completion. Our data were collected from October 2016 to December 2016 and included two phases. At time one, from October 15, 2016 to October 25, 2016, questionnaires were distributed to subordinates to measure empowering leadership, person-job fit, person-group fit and proactive personality and employees’ demographic information. At time two, 1 month later, questionnaires were distributed to subordinates to measure work engagement. We approached 12895 employees and obtained 10173 responses, yielding a response rate of 78.89%. Further, 3994 questionnaires were considered invalid due to omitted or incorrect answers. Finally, we obtained 6179 usable responses from 10173 responses, resulting in a usable response rate of 60.74%. Of the 6179 employees, 75.00% were male. The average age of the participants was 29.75 years (Range = 20–59, *SD* = 5.23). Among the 6179 employees, 1.36% had finished high school education or below, 87.57% had a Bachelor’s degree and 11.07% had a Master’s degree or above.

### Measures

All measures were rated on a five-point Likert-type scale ranging from 1 (strongly disagree) to 5 (strongly agree). Since empowering leadership was written initially in Chinese and other original scales were developed in English, all of the items underwent a back-translation process except empowering leadership (Brislin, 1986). All measures were completed by employees.

#### Empowering Leadership

Empowering leadership were measured using the 24 items scale from Wang et al. (2008). A sample item was “My manager gives me the authority I need to make decisions that improve work processes and procedures.” The Cronbach’s α for this scale was 0.938.

#### Person-Job Fit

Person-job fit was measured using the 4 items scale adopted from Saks and Ashforth (1997). An example of the items used was “To what extent do your knowledge, skills and abilities match the requirements of the job.” The Cronbach’s α for this scale was 0.811.

#### Person-Group Fit

Person-group fit was assessed using De Cooman et al.’s (2016) nine-item scale. The items were “My personality is similar to the team members I work with,” “My ability level is comparable to those of my team members” and “I feel that I am importance to this team because I have such different skills and abilities than my team members.” The Cronbach’s α for this scale was 0.807.

#### Work Engagement

Work engagement was assessed using the 9-items scale developed by Schaufeli and Bakker (2004). A sample item was “At my work, I feel bursting with energy.” The Cronbach’s α for this scale was 0.935.

#### Proactive Personality

Proactive personality was measured using Seibert et al.’s (1999) ten-item Proactive Personality Scale (PPS) (Seibert et al., 1999). A sample item was “I am constantly on the lookout for new ways to improve my life.” The Cronbach’s α for this scale was 0.843.

#### Control Variables

A number of control variables were included in line with previous research (Newman et al., 2017). Specifically, we used age, gender, education, rank and year as the control variables. Dummy variables were used to measure gender (1 = female, 2 = male), and education was coded as 1 for respondents who had a high school education or below, 2 for completing a college education, 3 for holding a master’s degree and 4 for holding a doctoral degree or above.

##### Analytical approach

Since our data was derived from individual-level, we used OLS regression to analysis the data and test H1. To test the two set of indirect effects proposed by H2 and H3. We used sample-based bootstrapping to conduct product of coefficient test. Product of coefficient approach is more effective in maintaining a balance between type 1 and type 2 error (Mackinnon et al., 2002). We using the PROCESS module 4 at SPSS (Preacher and Hayes, 2008) to test these indirect effects. To test the moderation effect proposed by H4, we grand-mean centered the predictor and moderator and then created interaction term. We then enter the interaction term into regression. To facilitate the result interpretation, we calculated the simple slope under high (+SD) and low level (-SD) of moderator (Aiken et al., 1991). Finally, H5 and H6 propose two set of moderated mediation effect. We employed Edwards and Lambert’s (2007) moderated path analysis approach to estimate the conditional indirect effects under high and low level of moderator. We used Mplus7.0 to draw the confidence interval derived from 1000 sample-based bootstrapping.

## Results

### Descriptive Statistics and Correlations

**Table 1** presents the means, standard deviations, and Pearson correlations of the measured variables. The correlations showed that empowering leadership was positively related to person-job fit (*r* = 0.59, *p* = 0.000) and person-group fit (*r* = 0.46, *p* = 0.000). Person-job fit was positively related to work engagement (*r* = 0.59, *p* = 0.000). Person-group fit was positively related to work engagement (*r* = 0.49, *p* = 0.000). Thus, the mediating role of person-job fit and person-group fit received initial support (Baron and Kenny, 1986).

**Table 1:** Means, standard deviations, and correlations for all included variables.

Variable	Mean	*SD*	1	2	3	4	5	6	7	8	9	10
(1) Gender	1.25	0.43										
(2) Age	29.75	5.23	0.01									
(3) Edu	2.10	0.35	0.05^**^	-0.05^**^								
(4) Rank	5.40	2.15	-0.00	0.67^**^	0.10^**^							
(5) Year	4.27	4.04	0.13^**^	0.64^**^	-0.14^**^	0.60^**^						
(6) EL	4.11	0.50	-0.05^**^	-0.07^**^	-0.03^*^	-0.04^**^	-0.07^**^	0.94				
(7) PP	3.94	0.51	-0.15^**^	-0.07^**^	-0.02	-0.05^**^	-0.10^**^	0.47^**^	0.84			
(8) PJFIT	4.05	0.61	-0.04^**^	0.01	-0.01	0.02	-0.01	0.59^**^	0.43^**^	0.81		
(9) PGFIT	3.76	0.50	-0.08^**^	0.07^**^	-0.01	0.12^**^	0.05^**^	0.46^**^	0.53^**^	0.48^**^	0.81	
(10) WE	4.15	0.60	-0.05^**^	0.01	-0.02	-0.01	-0.03^*^	0.59^**^	0.52^**^	0.59^**^	0.49^**^	0.94


*n = 6179. 1 = male, 2 = female; EL, Empowering leadership; PP, Proactive personality; PJFIT, Person-job fit; PGFIT, Person-group fit; WE, Work engagement. ^∗^p < 0.05, ^∗∗^p < 0.01. Education: (1) Middle school or below; (2) High school; (3) Bachelor; (4)Master; (5) doctor or above; Numbers in the parentheses are coefficient alpha.*

### Mediation and Moderation Effects

We performed OLS regressions using SPSS 20, and the results are presented in **Table 2**. Empowering leadership was positively related to employees’ work engagement (*β* = 0.60, *p* = 0.000, Model 2), thus hypothesis 1 was supported. To test the indirect effects, we conducted product of coefficient test using the PROCESS module 4 at SPSS (Preacher and Hayes, 2008), we used 1000 bootstrapping samples and then reported bias-corrected confidence intervals for each indirect effect. We summarize all the results in **Table 3**. Specifically, the indirect effects of empowering leadership on work engagement though person-job fit (95% CI = [0.19, 0.24]) and person-group fit (95% CI = [0.08, 0.14]) are significant. Thus, Hypothesis 2 and 3 are supported.

**Table 2:** Mediation effects.

Variables	Work engagement	
	
	Model_1_	Model_2_
Gender	-0.04^**^	-0.02
Age	0.05^**^	0.08^**^
Edu	-0.02	0.00
Rank	-0.01	-0.03^*^
Year	-0.05^**^	-0.02
EL		0.60^**^
*R*^2^	0.01	0.36
*F*	5.72^**^	565.91^**^
Δ*R*^2^	0.01	0.35
ΔF	5.72^**^	3351.35^**^


*n = 6179, ^∗^p < 0.05, ^∗∗^p < 0.01; (EL, empowering leadership; PJ, person-job fit; PG, person-group fit; WE, work engagement).*

**Table 3:** Bootstrap analysis of the magnitude and statistical significance of the direct and indirect effects.

Independent variable	Mediator variable	Dependent variable	Beta standardized direct/indirect effect	*SE* of mean	95 % CI [lower, upper]
EL		WE	0.71^∗∗^	0.01	[0.69, 0.74]
EL	PJ	WE	0.22^∗∗^	0.01	[0.19, 0.24]
EL	PG	WE	0.11^∗∗^	0.02	[0.08, 0.14]


*n = 6179; ^∗^p < 0.05, ^∗∗^p < 0.01; (EL, empowering leadership; PJ, person-job fit; PG, person-group fit; WE, work engagement).*

**FIGURE 2:**
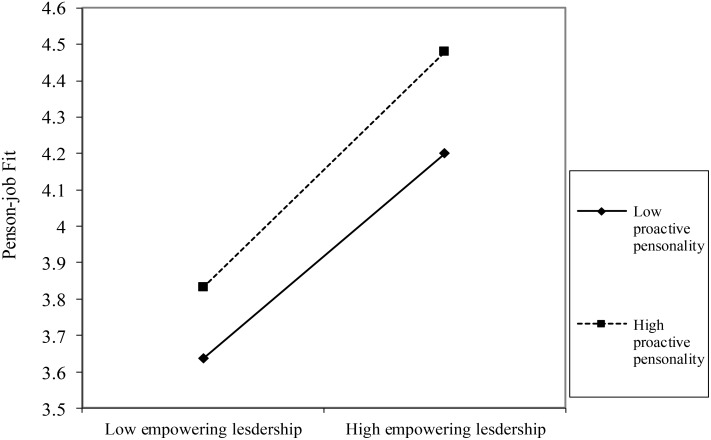
Interactive Effect of empowering leadership and proactive personality on person-job fit.

**FIGURE 3:**
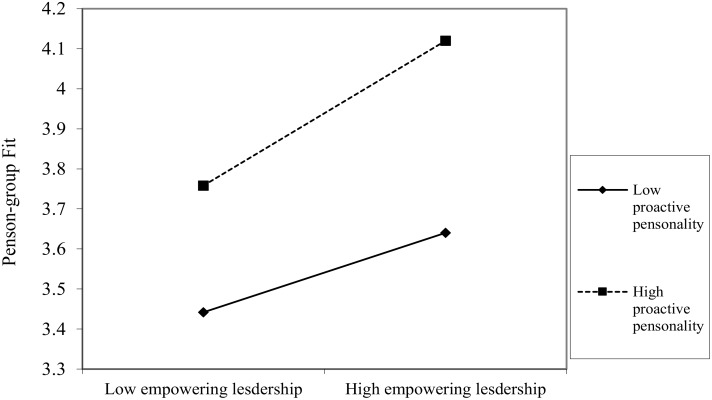
Interactive Effect of empowering leadership and proactive personality on person-group fit.

To test the moderation effect of proactive personality in the relationship between empowering leadership and employees’ work engagement, a moderated regression analysis was conducted by SPSS.20. As reported in **Table 4**, the interaction of proactive personality and empowering leadership were both significant in predicting person-job fit (*β* = 0.04, *p* = 0.000, Model 4) and person-group fit (*β* = 0.09, *p* = 0.000, Model 6). Further, the relationship between empowering leadership and person-job fit (**Figure 2**) was stronger for followers with high proactive personality (simple slope = 0.324) than for those with low proactive personality (simple slope = 0.282). The relationship between empowering leadership and person-group fit (**Figure 3**) was stronger for followers with high proactive personality (simple slope = 0.181) than for those with low proactive personality (simple slope = 0.099). Thus, Hypothesis 4a and 4b are supported.

**Table 4:** Moderation effects.

Variables	Person-job fit		Person-group fit	
		
	Model_3_	Model_4_	Model_5_	Model_6_
Gender	-0.04^**^	0.01	-0.08^**^	-0.01
Age	0.01	0.04^**^	0.00^*^	0.03^*^
Edu	-0.02	0.00	-0.02	0.00
Rank	0.04^*^	0.03	0.13^**^	0.12^**^
Year	-0.04^*^	0.01	-0.02	0.02
EL		0.50^**^		0.28^**^
PP		0.20^**^		0.40^**^
PP^∗^EL		0.04^**^		0.09^**^
*R*^2^	0.00	0.38	0.02	0.37
*F*	4.07^**^	468.78^**^	25.97^**^	456.37^**^
Δ*R*^2^	0.00	0.00	0.02	0.01
ΔF	4.07^**^	14.68^**^	25.97^**^	81.71^**^


*n = 6179; ^∗^p < 0.05, ^∗∗^p < 0.01. (EL, empowering leadership; PJ, person-job fit; PG, person-group fit; WE, work engagement).*

### Moderated Mediation Test

Hypotheses 5a and 5b predicted that proactive personality moderates the indirect effect of empowering leadership on work engagement via person-job fit/person-group fit. We tested these two hypotheses according to the moderated path analysis approach with 1000 bootstrapping samples (Edwards and Lambert, 2007) with software Mplus7. The results reported in **Table 5** supported the first-stage moderation effects, revealing significant moderating effects of proactive personality because the paths from empowering leadership to person-job fit differed significantly across different levels of proactive personality (0.08, *p* = 0.003) and the paths from empowering leadership to person-group fit differed significantly across different levels of proactive personality (0.16, *p* = 0.000). This provides additional support to our findings as to the moderating Hypotheses 4a and 4b reported in **Table 4**.

**Table 5:** Results of the moderated mediation.

Moderator variable:	Stage		Effect	
		
PP	First P_M1X_	Second P_Y M1_	Direct P_Y X_	Indirect P_M1X_ × P_Y M1_
**EL (X) → PJ Fit (M1) → WE (Y)**				
Low PP differentiation (-1 SD)	0.56^**^	0.33^**^	0.39^**^	0.16^**^
High PP differentiation (+1 SD)	0.64^**^	0.23^**^	0.26^**^	0.18^**^
Differenced between low and high	0.08^**^	-0.09^**^	-0.12^**^	0.02^**^
**EL (X) → PG Fit (M2) → WE (Y)**				
Low PP differentiation (-1 SD)	0.20^**^	0.16^**^	0.39^**^	0.03^**^
High PP differentiation (+1 SD)	0.36^**^	0.13^**^	0.26^**^	0.05^**^
Differenced between low and high	0.16^**^	-0.03	-0.12^**^	0.02^**^


*n = 6179; ^∗^p < 0.05, ^∗∗^p < 0.01. (EL, empowering leadership; PJ, person-job fit; PG, person-group fit; WE, work engagement).*

Regarding Hypothesis 5a, as shown by **Table 5**, the indirect effect of empowering leadership on work engagement via person-job fit was significant when proactive personality was low (*γ* = 0.16, *p* = 0.000) and high (*γ* = 0.18, *p* = 0.000). Furthermore, the difference of the indirect effects was significant (Δ*γ* = 0.02, *p* = 0.004). Thus, Hypothesis 5a was supported. The results in **Table 2** also indicate that the indirect effect of empowering leadership on work engagement via person-group fit was significant under high proactive personality (*γ* = 0.05, *p* = 0.000) and low proactive personality (*γ* = 0.03, *p* = 0.000). Furthermore, the difference in the indirect effects of proactive personality was significant (Δ*γ* = 0.02, *p* = 0.000). Hence, Hypothesis 5b was supported.

## Discussion

### Theoretical Implications

In the present study, we built and tested a model that explains the relationship between empowering leadership and work engagement. By doing this, our research makes three contributions.

First, our study is along the way to further link empowering leadership to employee work engagement. Although several researches have investigated relationships between leadership (e.g., transformational leadership, servant leadership, authentic leadership) and employee work engagement (Zhu et al., 2009; Aryee et al., 2012; Jorge Correia de Sousa and van Dierendonck, 2014), empowering leadership has been underrepresented (One exception is Tuckey et al., 2012). We diverge from Tuckey et al. (2012)’s work by using person environment fit framework. As a result, we expect the positive relationship between empowering leadership and work engagement, which will be transmitted through two separate fit perceptions. In addition, we specifically contribute to the engagement literature by demonstrating the importance of leadership in explaining the determinants of employee engagement.

Second, by examining the effects of P-G fit and P-J fit as mediators of the relationship between empowering leadership and work engagement, the present study contributes to both the leadership and the fit literatures. Specifically, we established task-related and relational-related fit perceptions as potential mediators by which empowering leadership transmits its effects (Kristof-Brown et al., 2005). By doing so, we provide a comprehensive understanding of how person-environment fit theory can invoke empowering leadership and work engagement literatures. Specifically, we contribute to the fit literature by demonstrating the connection of empowering leadership with not only person-job fit, but also person-group fit. Most prior empirical research have examined the effect of single fit perception (Seong and Choi, 2014; Pierro et al., 2015), few research has explored person-job and person-group fit jointly. Hence, we integrated them into a coherent model, wherein person-job fit serves as a task-oriented mechanism, and person-group fit serves as a relation-oriented mechanism.

Finally, this study further contributes to contextualized understanding of empowering leadership’s influence on various fit perceptions by investigating an important individual difference variable, proactive personality. We find that employees higher in proactive personality respond more positively to empowering leadership. More broadly, in order to understand which types of employees might respond more positively to empowering leadership, our study establishes the boundary conditions under which empowering leadership would be more effective. It also has important value on the growing research examining how the individual differences of employees influence their reactions to leaders (Antonakis et al., 2012).

### Practical Implications

Our findings also have several important managerial implications. First, our study indicates that empowering leadership, person-group fit and person-job fit have positive influence on employee work engagement. Thus, in order to enhance employee work engagement, managers need to pay more attention to the leadership behaviors. We recommend that managers should realize the positive relationship of empowering leadership on employee work engagement. More specifically, in recruiting practice, empowering leadership is an important consideration while enterprise managers make recruitment decisions. Besides, organization should provide team leaders with sufficient training about empowering leadership behaviors so that they can learn to delegate the authority, support and coach the followers and give them opportunities to participate in decision-making.

Second, we found that empowering leadership is effective in fostering employee work engagement through eliciting high-level P-G fit and P-J fit. In order to improve employees’ work engagement, leaders should pay more attention to foster employees’ perceptions of person-job fit and person-group fit through communication and socialization process in the team. Cable and DeRue (2002) purported that managers can affect employee’s person-job fit during both anticipatory socialization and after organizational entry. In the workplace, leaders can provide employees with appropriate support and guidance which focus on developing employees’ knowledge, skills, and abilities for meeting their job and organization’s demands, which may enhance employees’ perception of person-job fit and person-group fit (Guan et al., 2011; Bui, 2017).

Finally, our results suggest that employees high in proactive personality are found to respond more positively to empowering leadership in the form of higher person-job fit and person-group fit. Based on this, we would advise organizations to recognize the benefit of selecting more proactive employees than passive employees. Therefore, evaluating employees’ proactive personality is very important for understanding which employees would benefit more from empowering leadership. Furthermore, we suggest that leaders should also consider other characteristics which will influence their fit perceptions (Pierro et al., 2015). In addition, organizations should match followers’ proactive personalities with leaders’ behavior to maximize employees’ work engagement.

## Limitations and Future Directions

This study should be considered with several potential limitations, which in turn offer several suggestions for future research. First, because the data used in this study were collected from one source, which may cause potential common method variance. Although we collected the data at two time points, future studies should collect studied variables from different sources to strengthen causal inference. In addition, the date was from a single organization in China, which may influence our findings’ generalizability to other organizations and cultural contexts. To solidify the generalizability of our findings, replicate research should be exerted in different contexts. Notably, given our large sample size, it is relatively easier to detect significant relationships, so our empirical findings need more replication studies to further provide its theoretical implications.

Second, because our study did not control other leadership style, to ensure the increased variance that can be explained by empowering leadership, further researches could consider multiple leaderships together to decide the unique variance which could be attribute to specific leadership style.

Finally, our findings are limited to only one outcome, work engagement. Future research may examine a broader range of work behavior, especially performance-related outcomes, such as in-role and out-role performance. Besides, existing studies have analyzed team-level person-group fit and team outcomes, thus more works were needed to be done to examine the link between empowering leadership and team-level fit (Kristof-Brown et al., 2014; Seong and Choi, 2014). Therefore, we encourage future studies to explore the influence of empowering leadership on group level engagement.

## Ethics Statement

An ethics approval was not required as per the author’s Institution’s guidelines and national regulations. The CEO of the company supported this research. For the participants, we sent online questionnaire which directly links to individual members. They were completely free to join or drop out the survey. Only those who were willing to participate were recruited. The cover page of the questionnaire lay out the study objectives, the voluntary nature of the survey, and an assurance of confidentiality to the participants.

## Author Contributions

DC is responsible for idea generation, manuscript writing for theoretical part, and data collection. YC is responsible for idea generation, manuscript revision. YS is responsible for data analysis. JM is responsible for initial method part writing.

## Conflict of Interest Statement

The authors declare that the research was conducted in the absence of any commercial or financial relationships that could be construed as a potential conflict of interest.
